# Variability in performance of agility dogs navigating a dynamic obstacle

**DOI:** 10.3389/fvets.2024.1492391

**Published:** 2024-12-04

**Authors:** Arielle Pechette Markley, Moriah K. Wood, Abigail B. Shoben, Rachel A. Olson

**Affiliations:** ^1^Department of Veterinary Clinical Sciences, College of Veterinary Medicine, The Ohio State University, Columbus, OH, United States; ^2^Red Sage Integrative Veterinary Partners, Fort Collins, CO, United States; ^3^Department of Biology, The University of Akron, Akron, OH, United States; ^4^College of Public Health, Division of Biostatistics, The Ohio State University, Columbus, OH, United States

**Keywords:** agility, dog, teeter, sports performance, canine sports medicine, biomechanics, dog agility, seesaw

## Abstract

**Introduction:**

During agility performance, dogs complete a preset obstacle course. The teeter, also known as the seesaw, is the only dynamic contact obstacle. Dogs handle dynamic obstacles differently than static obstacles due to the need for increased coordination and postural control. No studies have been performed evaluating dogs’ abilities or biomechanical strategies to navigate the teeter. The goal of this study was to describe and quantify variability in teeter performance across a sample of dogs of differing body mass and breeds.

**Materials and methods:**

Twenty dogs of various body masses and breeds were recruited. Handlers were instructed to line their dog up approximately 5 m from the teeter and to handle the obstacle in a way to best reflect the dog’s typical performance. Repetitions were filmed using a GoPro Hero 11 at 240 frames per second. Data were post processed and footfalls were manually tracked using XMALab. Descriptive statistics were used to describe both central tendency and variability.

**Results:**

Mean total obstacle completion time (from dog breaking the plane of the teeter until teeter contact with ground) was 1.31 s (sd = 0.38) and mean total footfalls on the teeter was 18.3 (sd = 3.4). Footfall patterns varied across all phases of teeter performance, with particularly noteworthy variation during descent while the teeter was moving. Some dogs were nearly completely stationary while the teeter dropped while others continued to take steps toward the end of the obstacle as the teeter was in motion. Smaller dogs had more total footfalls and longer teeter completion times than larger dogs, and dogs with a stopped contact behavior took longer to fully exit the teeter after it contacted the ground.

**Discussion:**

These data imply that dogs use a variety of biomechanical strategies to perform a dynamic obstacle. Results of this study provide insight into teeter performance and variables that can be utilized for evaluation in future biomechanical studies. This study also provides initial data on biomechanical strategies used by dogs on dynamic surfaces, which may offer insight into dynamic stability and postural control in dogs and how that may influence injury development during sport.

## 1 Introduction

The canine discipline of agility is popular internationally, with over 1.2 million competition entries in American Kennel Club (AKC) sanctioned events alone in 2023 (1). During agility performance, dogs complete a course of obstacles in a pre-designated, specific order. These obstacles may include jumps, tunnels, weave poles, and contact equipment (A-frame, teeter, dog walk). Contact obstacles require the dog to enter on one end and exit the other end by placing at least a portion of one paw in the yellow “contact zone” at the end of the exit board. Agility is a test of both speed and training, with errors receiving faults or a time penalty, and the fastest time winning.

The teeter, also known as the seesaw, is the only dynamic contact obstacle. The teeter consists of a plank, usually made of fabricated material, though older designs used wood, which is typically coated in a rubber skin. This plank is supported at the center by a base that acts as a fulcrum (2). Equipment specifications vary by agility organization. In general, the teeter plank is 12 inches wide and 12 feet long and is required to have a non-slip surface. The height of the teeter is 24″ at the pivot point. For the AKC, the designated “contact zones” are 36 inches long and are colored in contrasting color from the remainder of the plank. AKC regulations require that the teeter is specifically designed so that it is balanced and hits the ground in less than 3 s when a 3-pound weight is placed 12 inches from the raised end (2). The event organizer must have on-hand the materials to correct a slow-dropping teeter (duct tape/fasteners, weights, etc.) (2). Dogs must ascend the plank and then cause the plank to pivot. In AKC, dogs must touch the “up” contact zone with any part of one foot, though other agility organizations do not have “up” contact zone requirements (2). For all agility organizations, at least one paw must touch the “down” contact zone after the plank has touched the ground prior to exiting the obstacle with all four paws (2). The dog must exit the descent end of the teeter. Standard faults (point/time deductions) are given if the dog misses the up (in AKC) or down contact zone, or if the dog jumps off the end of the teeter plank before the board contacts the ground (called a “fly-off”) (2).

**Figure 1:**
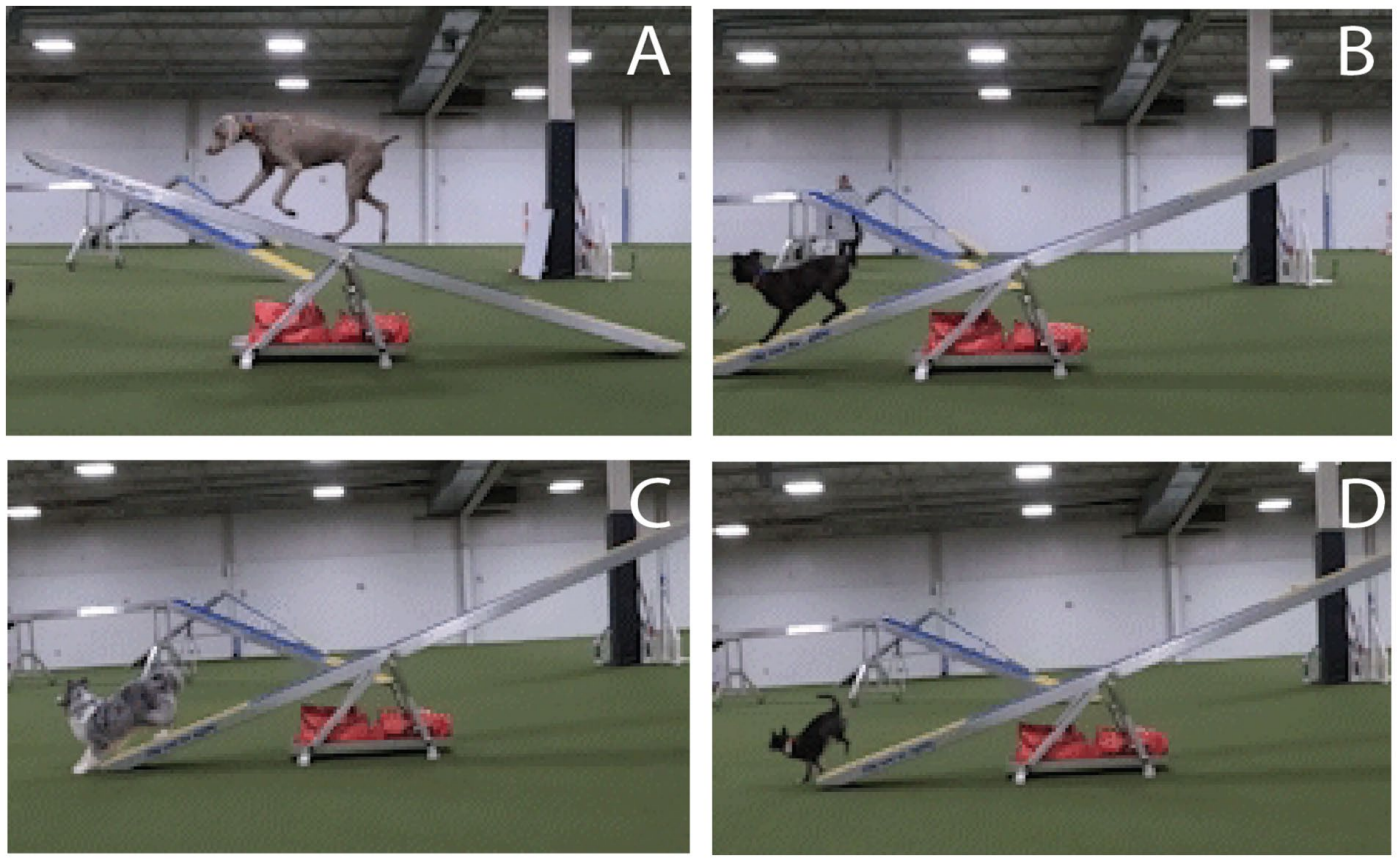
Images of teeter performance. **(A)** A dog crossing the pivot point of the teeter, **(B)** a dog performing a “all four on standing” (4o) behavior, **(C)** a dog performing a “two-on two-off” (2o2o) behavior, and **(D)** a dog performing a running contact.

A variety of training strategies are employed for this obstacle. To ensure successful completion of the obstacle, where the teeter touches the ground and the dog has at least one paw in the “down” contact, many handlers train the dog to perform a specific behavior at the end of the teeter, also called a contact behavior (Figure 1). The two most commonly trained behaviors include a “two-on two-off” (2o2o; Figure 1C) and an “all four on standing” (4o; Figure 1B), with the overwhelming majority performing a “two-on two-off” behavior (3). The 2o2o behavior is where the dog is trained to run to the end of the plank, place the two front feet on the ground off the plank while keeping the rear two feet on the down contact. Typically, the dog is trained to remain in that position until verbally released. A 4o behavior is where the dog is trained to run to the end of the plank and stop with all 4 paws on the plank as close to the end as possible, either in a standing position (more common), a down or a bow position (less common) and the position is typically held until released by the handler. A running contact is also performed by some dog-handler teams, where no stop is performed after the plank contacts the ground, and requiring no release, although the dog is often stationary during descent (Figure 1D). Some handlers train with a stationary contact behavior (2o2o or 4o) but will do a quick/early release, where typical contact criteria are not upheld in exchange for speed, or running contact during major competitions. Methods for training these various contact behaviors vary. A successful performance reflects the dog’s physical, and mental, ability to compensate for the dynamic obstacle movement. A failure to successfully perform this obstacle will result in the best case, a time penalty, and in the worst case, injury to the dog.

Injuries are common in agility dogs, with some studies reporting up to a 42% injury rate (4). Shoulder, iliopsoas muscle, digit and lower back injuries were most commonly reported (4). While other studies have evaluated possible risk factors for injury, minimal clear correlations have been observed. The most consistent correlations across studies have been increased risk of injury with Border Collie breed, higher competition level, less handler experience, and increased dog weight compared to height (3, 5–10). There have been very few correlations found between injury and specific obstacle performance. However, one study found that dogs who completed training for teeter contact behavior at a younger age had a lower risk of injury (3). The reasons for this were unknown, but one of the hypotheses was that dogs who were able to learn to negotiate the teeter quickly had better balance and coordination compared to dogs who took longer to learn to navigate this dynamic obstacle. In humans, increased balance and coordination has been shown to be associated with decreased risk of injury in athletes (11).

There has been much discussion among the agility community about how specific obstacles, performance techniques, and contact behaviors for those obstacles might influence injury risk. However, to date, there have been no studies specifically evaluating these factors. A study by Cullen et al., asked handlers in a retrospective survey if they thought a specific obstacle was associated with their dog’s injury (12). Commonly reported perceived causes of injury included direct contact with a bar jump and contact with/fall from an A-frame or dog walk (12). Based on these concerns, some biomechanical studies have been performed looking at kinetics and kinematics of jumping and A-frame performance, but none have looked at specific paw placement patterns, contact behaviors, or relationship to injury (13–26). Other video-based studies have aimed to look at paw placement patterns in the performance of obstacles such as weave poles and the dog walk with the intent of categorizing performance strategies to enable more in-depth studies. Those studies found that weave pole performance could generally be classified into 5 specific techniques, but that dog walk performance was too variable for classification (27, 28). However, due to the unique, dynamic nature of the teeter obstacle, studies evaluating other obstacles cannot be extrapolated to the teeter. Currently, no studies have been performed evaluating performance strategies, kinetics, or kinematics of the teeter obstacle.

The dynamic nature of the teeter obstacle makes it a unique obstacle to navigate. No studies have been performed evaluating dogs’ abilities or biomechanical strategies to navigate dynamic obstacles. A recent study evaluated the effect of external mechanical perturbations using a motorized training platform on a dog’s postural stability (29). Center of pressure was used to measure postural stability and, not surprisingly, it was found that external mechanical perturbations created a challenge for postural stability. They also found that an increase in amplitude of the perturbations created a greater challenge for postural stability than an increase in speed of the perturbations (29). This study noted that dogs did not tolerate the highest intensities of amplitude and speed in combination. It is unknown if or how a mechanical platform correlates with the movement and postural control needs for performance of the teeter obstacle. Other studies in dogs have evaluated how aging affects postural control, and how orthopedic surgery affects balance (30–32). However, neither of these populations are particularly relevant to the canine athlete population navigating dynamic obstacles. There is a very large body of research on dynamic stability and postural control in humans, in a variety of demographic populations including athletes. However, results are likely to be different based on bipedal versus quadrupedal biomechanics.

The goal of this study was to describe and quantify variability in different teeter performance strategies across a convenience sample of dogs of differing body mass and breeds, and to identify areas of interest for future biomechanical studies.

## 2 Materials and methods

### 2.1 Data collection

Twenty dogs without breed or size restriction were recruited for this study. All dogs were owner-reported to be competing at the Masters level of AKC agility (or equivalent) and to be free of orthopedic health conditions. The owner also reported breed, height at the withers, and mass.

The training facility was a 12,000 square foot, indoor, climate-controlled building used almost exclusively for dog agility training and competitions. The footing was GrassTex turf (product PL307). The teeter was a “Clip and Go Seesaw” which is an engineered rigid aluminum plank with a metal MAX/composite board top on the plank and a wet-pour UV-stable rubber surface. It has speed-limiting, cushioning cylinders on base to reduce board whip and rebound (cylinders are sealed), nylon pivot bushings on base, and energy-absorbing foam underneath the grip pads on the descent side of plank to cushion impact. The teeter was secured with sandbags on the fulcrum to limit extraneous movement, as is common in agility competitions. The drop rate of the Clip and Go Seesaw in the standard 3 lb.-weight test ranges from 1.6 to 1.9 s (33).

Handlers were instructed to line their dog up approximately 5 m away from the teeter and to handle the obstacle in a way of their choosing to best reflect the dog’s typical performance. Dogs were asked to perform a total of four repetitions of the teeter, two each with the handler on the left and right sides of the dog. Only a single repetition of the obstacle was performed per recording sequence. Dogs were filmed using GoPro Hero 11 at 240fps in linear mode while performing the obstacle.

All protocols were approved by the Ohio State University’s institutional animal care and use committee (IACUC #2022A00000058).

### 2.2 Data processing

Data was post-processed and footfalls were manually tracked using XMALab (version 2.1.0) for a single repetition for each dog with the handler on the right, so as to not obscure the dog’s performance. For each footfall, the position on the obstacle/ground and the duration of contact with the obstacle/ground (i.e., duty factor) was recorded. For positional footfalls, four areas of interest on the teeter were defined: the up-contact zone, the area past the up-contact but prior to the midpoint, the area past the midpoint but prior to the down contact zone, and the down contact zone. Per AKC rules, contact with any portion of the paw within the yellow contact zone (up or down) was considered a footfall within the contact zone. Total footfalls on the teeter were also recorded.

For the purpose of defining performance strategies, the following phases of teeter performance were described: 1. Approach; 2. Ascent; 3. Tip; 4. Descent; 5. Exit. Approach was defined as the stride before any contact with the teeter. Ascent was defined as the time between the dog’s nose breaking the plane of the teeter and when the teeter started to move. Tip was defined as the instantaneous moment the teeter began to move. Descent was defined as the time between when the teeter began to move to when it contacted the ground. Exit was defined as the time from when the teeter contacted the ground to the stride after all paws have left contact with the teeter. This may include a stationary period where the dog is holding a contact behavior.

Duty factor footfalls were counted within ascent, descent, and exit. When counting footfalls in these time intervals, a footfall held over two time intervals (e.g., both the ascent and descent) was classified based on where it was held longer. For these footfalls, resetting of paws were counted as a single footfall when they were not visually distinct on a duty factor plot. Note that footfalls on the ground before and after dog contact with the plank were not counted as total footfalls, but footfalls on the ground during exit (i.e., as part of a stationary contact behavior) were counted as duty factor footfalls.

Contact behaviors were defined as “stopped” or “not stopped.” A dog that had all four paws simultaneously stationary with at least one paw on the teeter after the teeter had contacted the ground was considered to have a “stopped” contact. The stopped contact behavior was further classified based on how many paws remained on the teeter while stopped: “4 on” (4o) if all four paws were on the teeter, “2 on 2 off” (2o2o) if the front paws were off and rear paws were still on, and “3 on 1 off” (3o1o) if 3 paws were on the teeter and one front limb was off. Sliding was not observed in any of the dogs in this study.

“Total time to completion” was defined as the time from when the dog’s nose broke the plane of the teeter until the teeter first contacted the ground (i.e., ascent time + teeter descent time). “Time to dog exit” was defined as the time from when the teeter first contacted the ground until no more paws were in contact with the obstacle. Additionally, “dog time to descent” was defined as the time from when the nose crossed the midpoint until the teeter touched the ground, whereas “teeter time to descent” was defined as the time from when the teeter started to move until it touched the ground (i.e., “descent” phase above). All times were calculated by counting the number of frames and converting to seconds.

### 2.3 Data analysis

Descriptive statistics were used to describe both central tendency (means) and variability (standard deviations, range). Dogs were grouped into 4 mass categories (<10 kg, 10–20 kg, 20-30 kg, and > 30 kg). Mass was chosen for categorical representation of dog size, as teeter descent is dependent on mass past the pivot point. Exploratory associations between dog mass and teeter performance variables were quantified with linear regression models. Statistical analysis and plots were performed in RStudio (version 2023.12.0 + 369) using the packages proxy (version 4.3.2), spatstat.geom (version 4.3.2), tidyr (version 4.3.2), and plotly (version 4.3.2).

## 3 Results

### 3.1 Overall teeter performance

The 20 participating dogs were a variety of breeds and sizes (full raw data available in Supplementary Tables 1, 2). The most common breed was the Border Collie (*n* = 6), but the sample also included four Labrador Retrievers, three mixed breed dogs, and three Weimaraners. The mean mass was 20.5 kg (sd = 8.9) with the smallest being a 4.0 kg Italian Greyhound and the largest a 39.6 kg Weimaraner. A stopped contact was observed for 13 of the 20 dogs (65%). The remaining dogs did not have a stopped contact, but a variety of “not stopped” behaviors were observed. The two smallest dogs (<10 kg) did not have a stopped contact and the two largest dogs (>30 kg) both did have a stopped contact.

**Figure 2:**
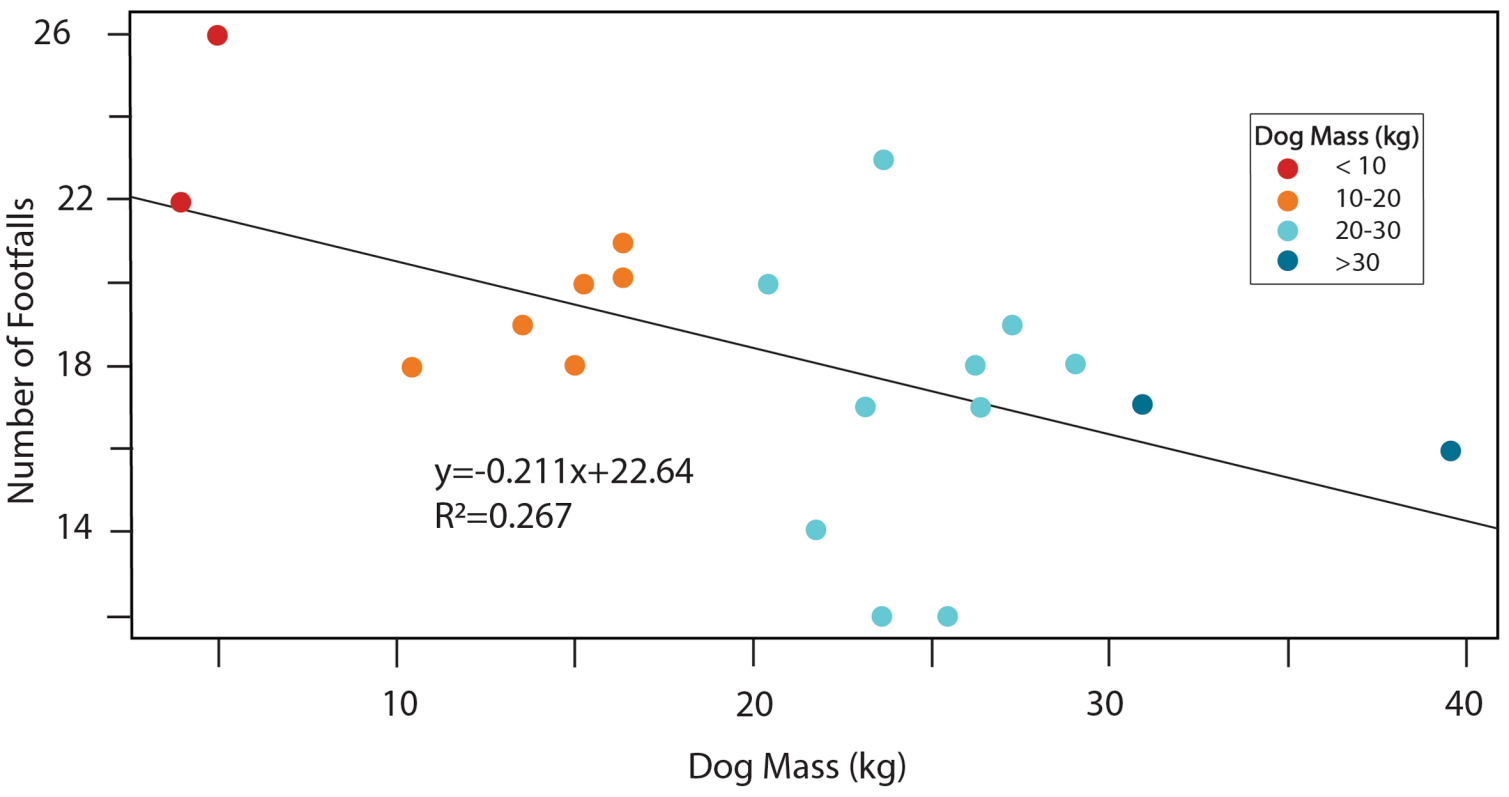
A relationship between dog body mass and the total number of footfalls was observed, with a trend of larger dogs having fewer footfalls than smaller ones (*p* = 0.012). Color coding indicates mass categories.

**Figure 3:**
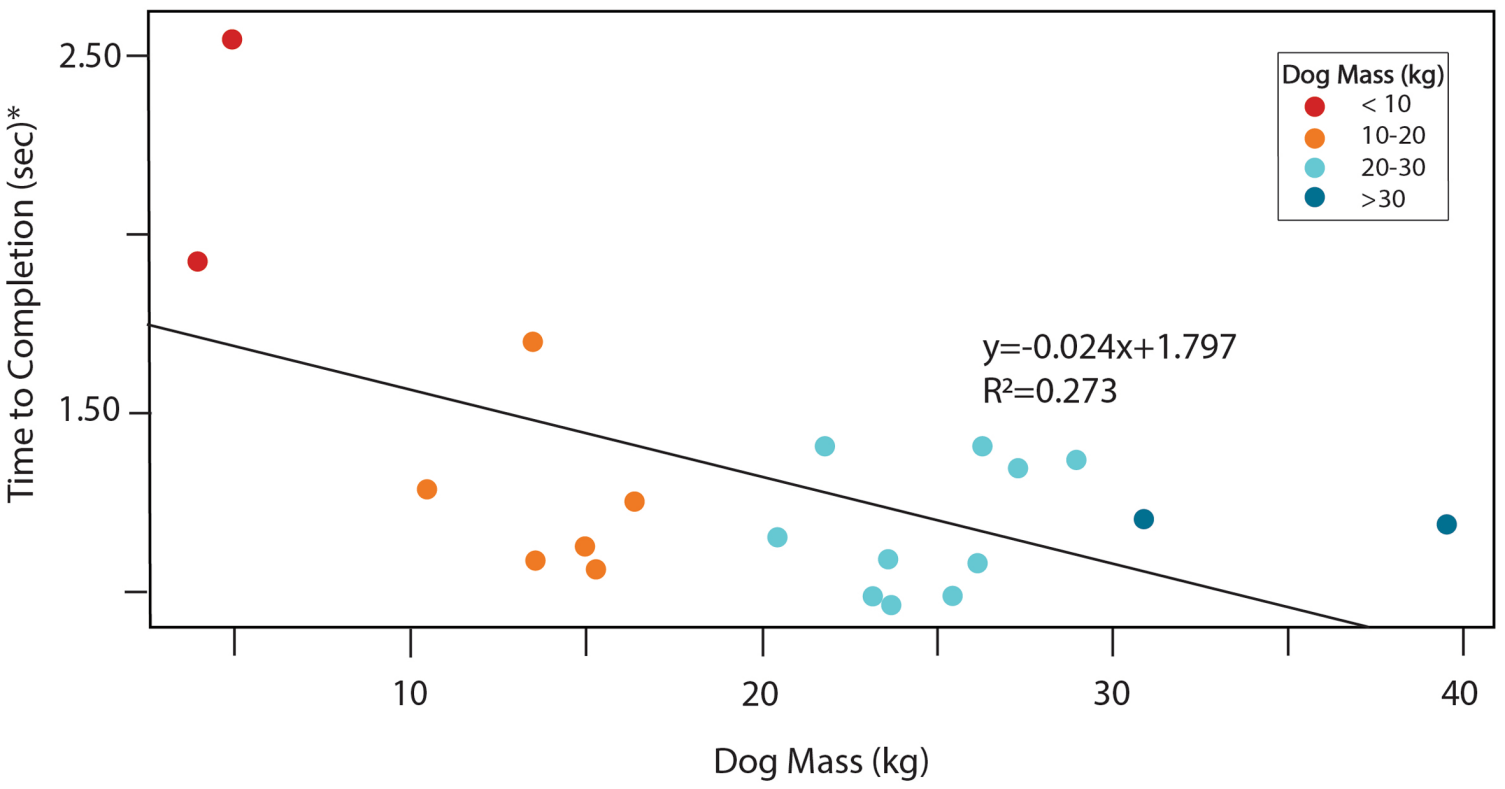
Scatterplot with superimposed linear regression line showing the association between dog mass and total obstacle completion time* (*p* = 0.011). Color coding indicates mass categories.

**Figure 4:**
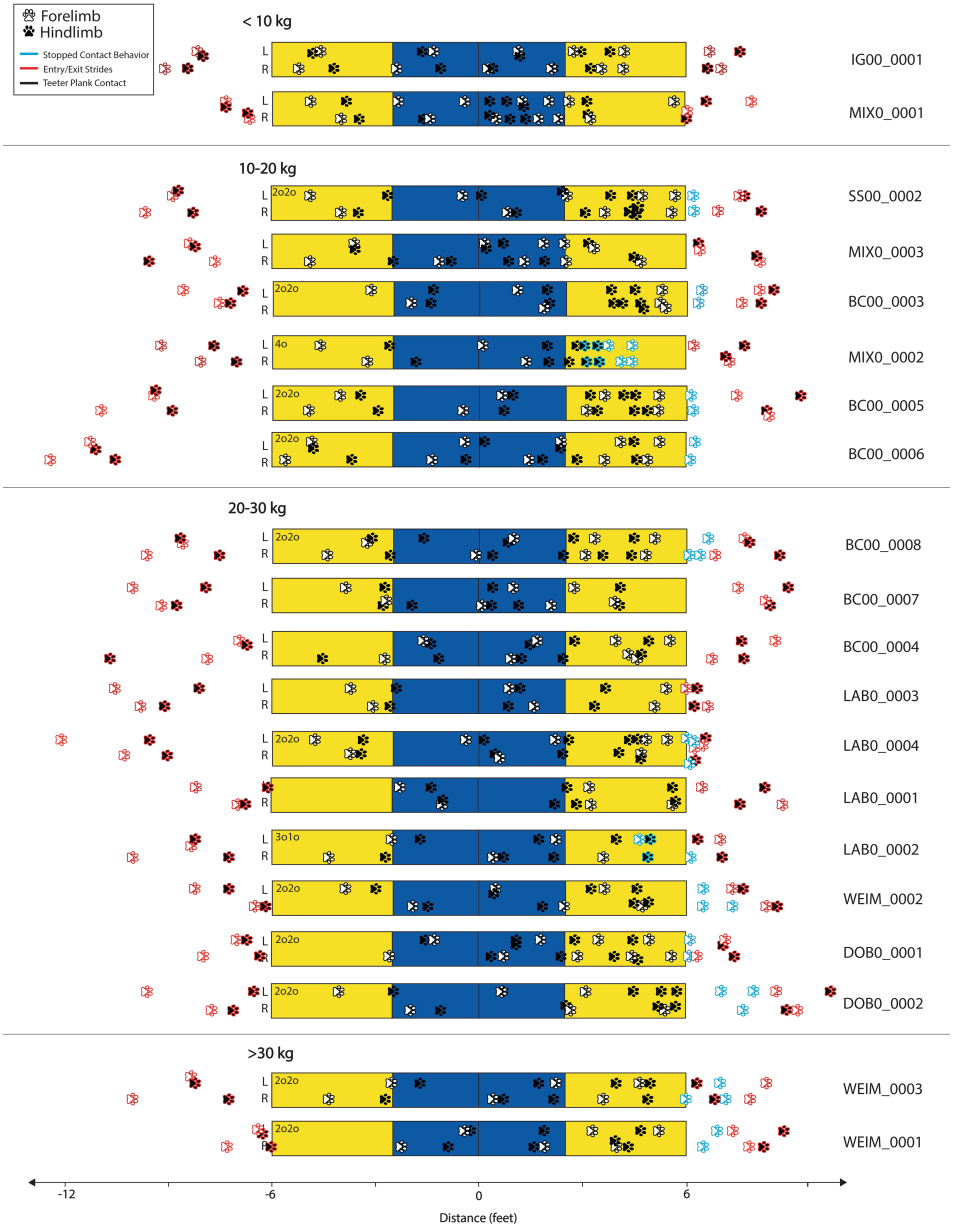
Paw position on the teeter obstacle for all 20 dogs. Front paws are shown with white fill and rear paws are shown with black fill. The strides before and after are shown in red and observed stopped contact behaviors are shown as blue outlines (2o2o, 4o, 3o1o), indicating that the dog was stationary after the teeter touched the ground. Dogs are separated by mass category (<10 kg, 10–20 kg, 20–30 kg, >30 kg). BC00_0006 held 2o2o stopped contact on video end.

**Figure 5:**
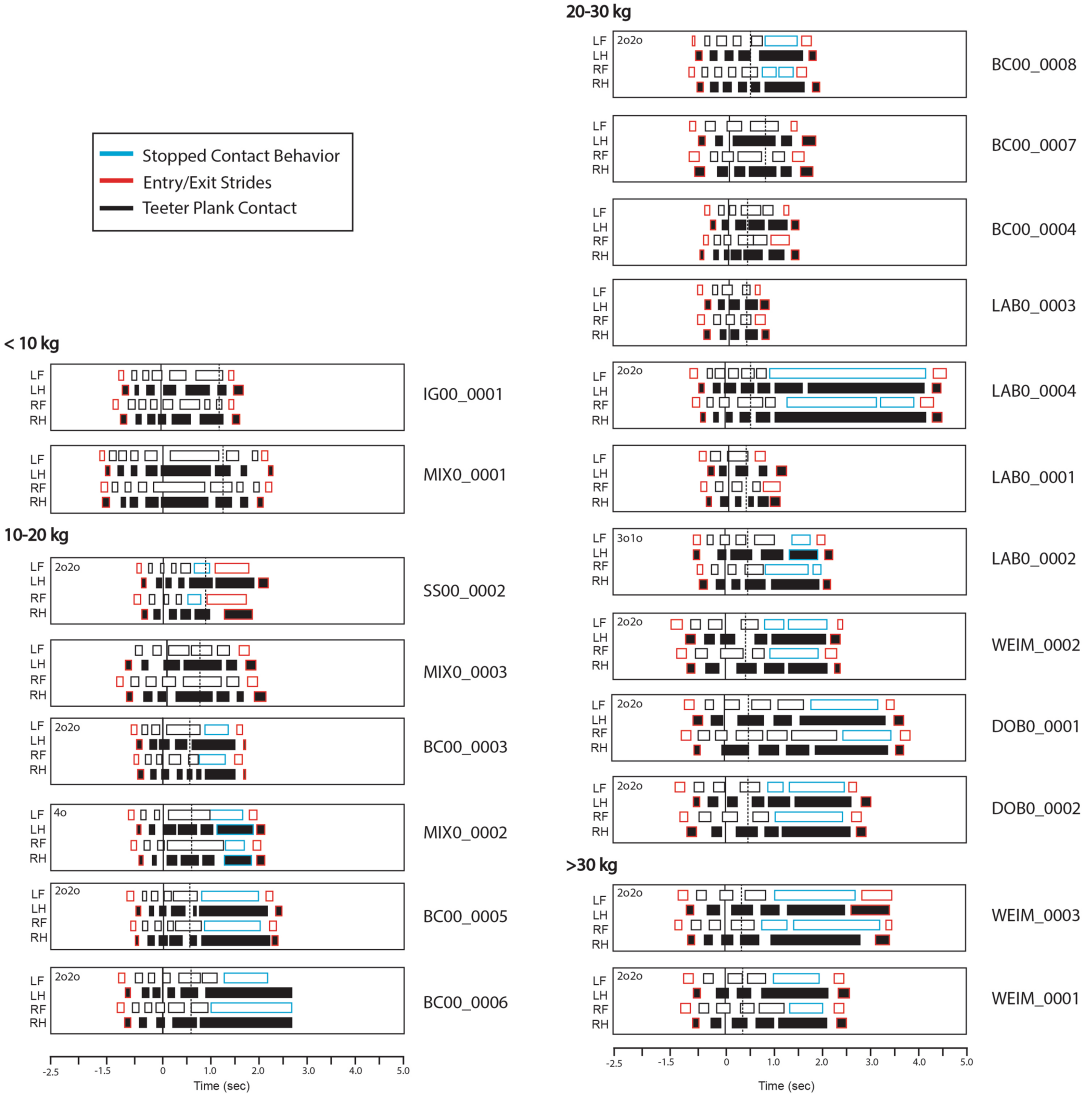
Timing, or duty factor, of paw contacts relative to teeter movement phase for each of the 20 dogs. Longer rectangles indicate longer paw contact times. Time = 0 when the teeter starts moving (depicted with a black line). Time of teeter contact with ground is depicted with a dashed line; therefore, the time between these lines is the descent period when the teeter is moving. Front paws are shown in white fill and rear paws are shown in black. Red outlines denote the stride before and stride after the teeter. Blue outlines indicate an observed stopped contact behavior (2o2o, 4o, 3o1o). Dogs are separated by mass category (<10 kg, 10–20 kg, 20–30 kg, >30 kg). BC00_0006 held 2o2o stopped contact on video end.

The mean total time for obstacle completion was 1.31 s (sd = 0.38), with a minimum of 0.96 s and a maximum of 2.55 s observed (Table 1). Total obstacle completion times were generally similar between dogs with stopped contacts and those without (Table 1). The mean number of duty factor footfalls was 18.3 (sd = 3.4), with a minimum of 12 and a maximum of 26, and these means were also similar between dogs with and without stopped contacts (Table 1). Dog mass was strongly associated with both total footfalls and obstacle performance times. Larger dogs had fewer total footfalls (Figure 2; *p* = 0.012). Larger dogs also had faster overall total obstacle completion times (Figure 3; *p* = 0.011). Paw positions on the teeter plank for all 20 dogs are shown in Figure 4 and the corresponding duration of each footfall (duty factor) is illustrated in Figure 5.

**Table 1:** Summary statistics for obstacle performance time in seconds and number of observed footfalls in each teeter phase.

	All dogs^°^ (*n* = 20)	Min, Max	Stopped^°^ (*n* = 13)	Not stopped^°^ (*n* = 7)
Overall				
Total obstacle completion time^*^	1.31 (0.38)	0.96, 2.55	1.21 (0.12)	1.50 (0.60)
Total number of footfalls	18.3 (3.4)	12, 26	18.8 (1.9)	17.4 (5.3)
Ascent				
Ascent time^‡^	0.61 (0.22)	0.32, 1.11	0.58 (0.18)	0.68 (0.28)
Ascent number of footfalls	6.2 (2.2)	4, 13	5.6 (1.2)	7.1 (3.2)
Descent				
Teeter time to descent^†^	0.70 (0.23)	0.51, 1.44	0.63 (0.12)	0.83 (0.33)
Dog time to descent^#^	0.90 (0.31)	0.65, 1.93	0.80 (0.10)	1.07 (0.48)
Descent number of footfalls	5.9 (2.4)	2, 12	5.6 (2.7)	6.4 (1.9)
Exit				
Time to dog exit^^^	1.24 (0.92)	0.02, 3.47	1.75 (0.77)	0.37 (0.23)
Exit number of footfalls	6.3 (2.9)	1, 11	7.5 (2.6)	3.9 (1.6)

* Time from when nose crosses teeter threshold until teeter touches the ground.

‡ Time from when nose crosses teeter threshold until teeter starts to move.

† Time from when teeter starts to move until teeter touches the ground.

# Time from when nose crosses midpoint until teeter touches the ground.

^ Time from when teeter touches ground to last paw contact (*n* = 19 total; 1 stopped contact still held on video end).

° Mean (SD).

### 3.2 Approach

Dogs appeared to show some variability in their entry stride into the teeter (Figures 4, 5). Some individuals (WEIM_001, DOB0_0001) had all four footfalls within a relatively small space on the ground close to the teeter, indicating more of a collection-type stride. Others (e.g., BC00_0006, LAB_0004) took the entry stride from further away and had a longer stride length, indicating greater relative extension. All dogs appeared to have average-to-short contact duration with the ground during the entry stride, as compared to their footfalls while on the teeter (Figure 5).

### 3.3 Ascent

Mean time for ascent was 0.61 s (sd = 0.22), with all dogs initiating the tip within 1.11 s. Initial paw positioning was variable during teeter ascent, with two dogs not placing any paws in the up-contact zone and nine individuals placing all four paws at least once in this region (Figure 4). Mean total number of footfalls during ascent was 6.2, with slightly more footfalls observed during ascent for dogs without stopped contacts (7.1 footfalls) than dogs with stopped contacts (5.6 footfalls). However, mean footfalls during ascent were virtually identical between the 13 dogs with stopped contacts, all of whom had a mass > 10 kg, and the 5 dogs with mass > 10 kg who did not have a stopped contact. The footfalls during ascent were generally short in duration relative to the footfalls during descent (Figure 5), although for some individuals there was an observed increase in contact time just prior to the tip point as well (e.g., MIX0_001).

### 3.4 Tip

**Figure 6:**
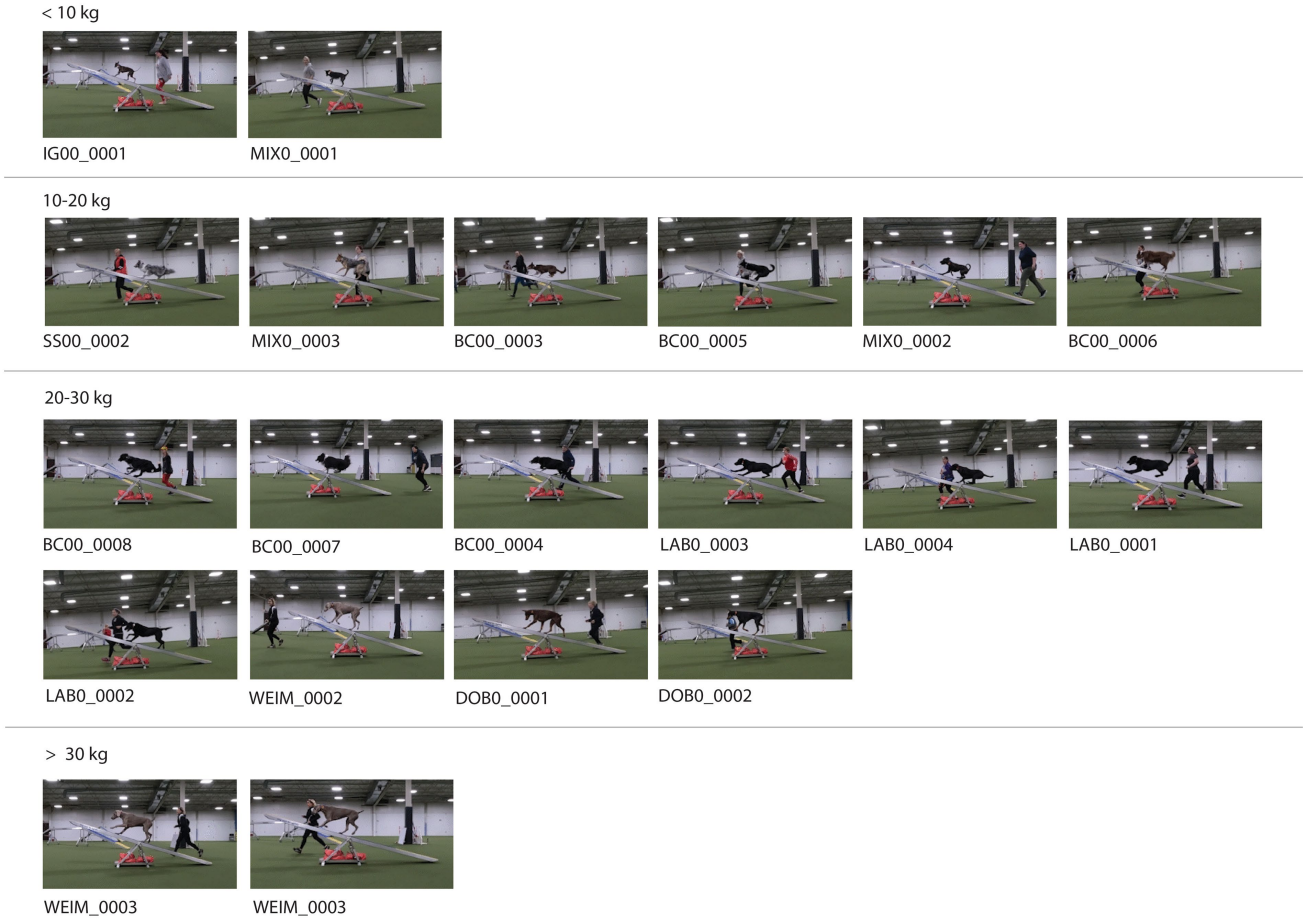
Image stills of all 20 dogs at the frame of initial teeter tip.

The location of the dog when the teeter started to move is shown in Figure 6. Some dogs initiated the tip near the midpoint of the teeter while others took a stride spanning the midpoint and initiated contact further along the plank. This variation was seen even within dogs of the same breed (e.g., LAB0_0001, LAB0_0002).

### 3.5 Descent

**Figure 7:**
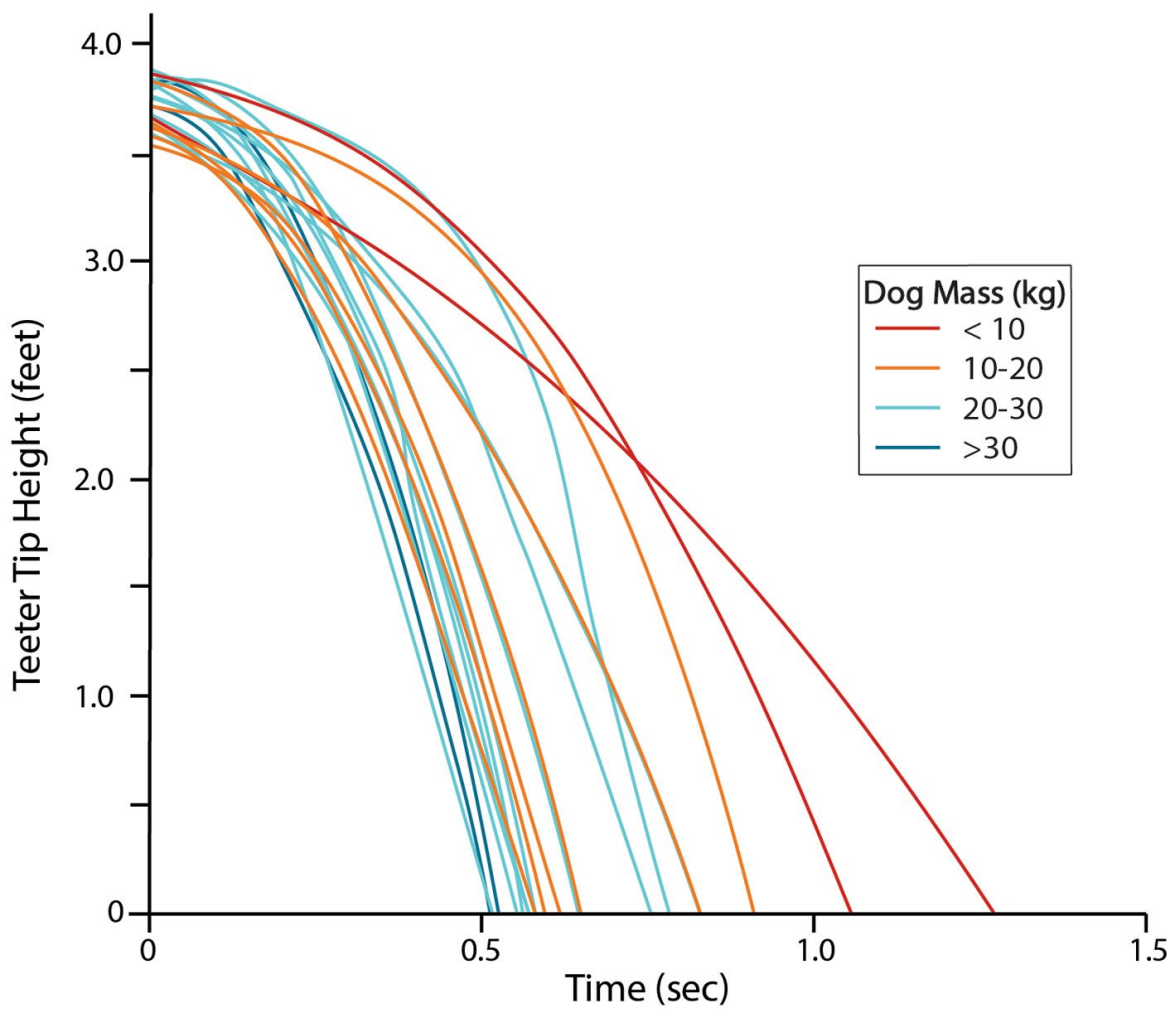
Descent of the teeter tip is faster in dogs of higher body mass. Color coding indicates mass categories.

**Figure 8:**
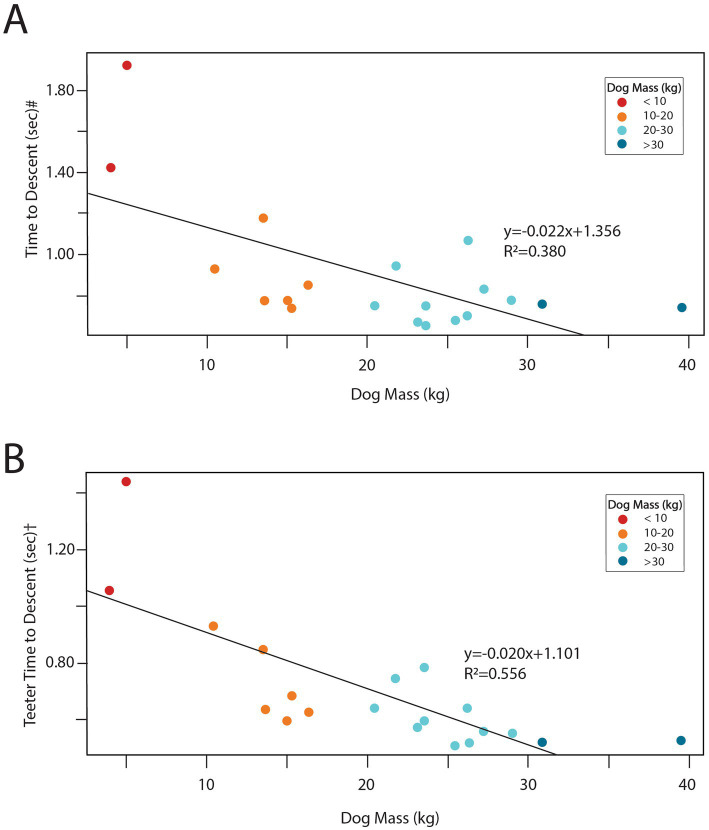
Scatterplots with superimposed linear regression line showing the association between dog mass and **(A)** dog time to descent# (*p* = 0.002), and **(B)** teeter time to descent† (*p* < 0.001). Color coding indicates mass categories.

The mean time for dog descent was 0.90 s (sd = 0.31, Table 1) and the mean time for teeter descent was 0.70 s (sd = 0.23, Table 1). The fastest teeter descent time was 0.51 s, with multiple dogs approaching 0.5 s (Figure 7). 15 of 20 dogs (75%) had times less than 0.75 s (Figure 7 and Supplementary Table 2). Descent times were longer for the smallest dogs with statistically significant associations observed between mass and dog descent time (Figure 8A; *p* = 0.002) and dog mass and teeter descent time (Figure 8B; *p* < 0.001).

Dogs appeared to use a variety of biomechanical strategies to navigate the moving plank during descent (area between the solid and dashed lines in Figure 5). Some dogs maintained a near-stationary position with long duty factors (e.g., MIX0_0001, MIX0_0003, BC00_0007), while others took multiple steps while the teeter was in motion (e.g., IG00_0001, SS00_0001). Often, larger dogs straddled the pivot point of the teeter during descent while smaller dogs stood further past the midpoint until the teeter contacted the ground, as seen from the still images in Figure 6 and the corresponding paw timings in Figure 5.

The mean number of footfalls during the descent (5.9, sd = 2.4) was similar to the mean number of footfalls during ascent (6.2, sd = 2.2) and generally similar between dogs with stopped and not stopped contacts (Table 1). All dogs contacted the down contact zone with multiple paws in this sample (Supplementary Table 1).

### 3.6 Exit

Of the 13 dogs with a stopped contact, 11 (85%) were classified as a 2o2o contact. In this controlled environment, dogs held stopped contact behaviors for varying amounts of time with a mean time to exit of 1.24 s (sd = 0.92) among dogs with stopped contacts (Table 1). One dog (BC00_0006) did not exit the obstacle during the video and was still holding a 2o2o.

Dogs with a stopped contact had more footfalls during the exit phase as compared to dogs without a stopped contact (Table 1). Some dogs were observed shifting their weight or taking small steps while maintaining the same contact behavior (e.g., the multiple blue footfalls in Figure 4: for DOB0_0002 and WEIM_0002).

The exit stride, or the first stride off the teeter, showed high variability in paw positioning, as illustrated on Figure 4. Some of this variability appears to be related to contact behavior, with dogs who had a 2o2o stopped contact placing their paws further from the exit edge of the teeter plank.

## 4 Discussion

The goal of this study was to describe and quantify variability in different teeter performance strategies across a sample of dogs of differing body mass and breeds and to identify areas of interest for future biomechanical studies. Dogs exhibited a substantial amount of variability in paw positioning, number of footfalls, duty factor, and obstacle performance times. There was considerable variability in biomechanical strategies for each phase of the teeter and notable differences in performance observed between the smallest and largest dogs.

### 4.1 Approach

Observationally, there was substantial variation in entrance strides between dogs, particularly with regards to the degree of collection and extension (i.e., relative stride length) exhibited upon entrance to the teeter (Figure 4). Visually, some of the Border Collies and Labrador Retrievers in this study entered the teeter with more extension compared to others of the same breed. Similarly, some Weimaraners, Doberman, and mixed dogs appeared to enter the teeter with more collection compared to others in their mass category. This variation may be a result of training methods or breed behavior, as this did not appear to be associated with mass. However, this study evaluated obstacles independently, with a straight-line approach, and not in sequence. Agility courses will have varying angles of approach to the teeter, as well as variations in speed of approach based on the previous obstacle type, orientation and the distance from the previous obstacle to the teeter. These variations in course design will likely affect the approach performance variables such as the degree of collection or extension. Approach stride lengths were not quantified as dogs started a relatively short distance from the teeter obstacle from a stationary position which is not representative of obstacle completion during typical agility obstacle performance. Therefore, these observations were based on visual estimations and were not corrected to actual stride lengths. Additional studies would be needed to evaluate entrance stride kinematics and relation to breed, conformation, training, and course design.

### 4.2 Ascent

Footfall placement and number of footfalls during the ascent phase was quite variable between dogs. Two dogs did not place any paws in the up-contact zone. In AKC, where the up-contact zone is judged, this would be considered a fault, though these two dogs questionably placed toes on the edge of the plank in the up-contact, so it may not have been judged as a fault. Some dogs placed a single paw in the up-contact zone but almost half of dogs had a whole stride (placement of all four paws) within the up-contact zone. The paw placements within the up-contact zone were correlated with dog size, with smaller dogs more likely to complete a whole stride in the up-contact. The two dogs that did not place a paw in the up-contact zone were larger dogs. These variations are likely a direct result of dog stride length, though training may also influence ascent striding.

When comparing the ascent variables between dogs with stopped and not stopped contact behaviors, there were no notable differences. This suggests that dogs may not have differences in preparation for the moving descent of the teeter based on trained contact behavior, though definitive conclusions cannot be made with this small sample size. The footfalls during ascent were generally short in duration relative to the footfalls during descent which may be related to the increased stability of the plank ascent compared to the dynamic movement of the plank descent. One dog appeared to significantly increase contact duration during the last few steps of ascent, potentially anticipating teeter movement (Figure 5: MIX0_001). Fear of teeter movement and the resulting noise on ground contact is a commonly encountered training challenge in dog agility. Future work that addresses the dog’s training history would provide insight if there are anticipatory-related effects on performance and if there are differences in obstacle performance in individuals who have had challenges with training compared to those who did not show aversion to movement during training. Variation in ascent performance may also be reflective of the highly variable approach stride and starting distance from the teeter. It is unknown how ascent variables may be affected by teeter placement within a course, which obstacle is placed prior to the teeter, the distance between the obstacles, and the line to approach.

### 4.3 Tip

The location of the dog’s torso at the moment of the teeter tip varied substantially. The teeter is a type of lever that consists of a flat surface and a center fulcrum. The force required to drop the teeter must overcome the mass of the portion of the plank in contact with the ground. Mass on the elevated side of the teeter increases the force, causing the teeter to drop. This force is larger with larger mass and when the mass is further from the fulcrum (i.e., longer lever arm). Thus, smaller dogs must move farther out onto the teeter to achieve enough force to overcome the mass of the plank compared to the required distance for larger dogs. Similarly, for dogs equally far away from the fulcrum, larger dogs will exert more force, causing the teeter to drop faster. The smallest dogs appeared to have their whole torso past the tipping point to overcome this inertia. Dogs greater than 10 kilograms often straddled the tipping point. In the larger dogs, some only crossed the tipping point with the head and forelimbs and yet were able to produce enough force to initiate movement.

### 4.4 Descent

Dogs appeared to use a variety of biomechanical strategies to navigate the moving plank during descent. Some dogs remained in a stationary position past the tipping point of the teeter as the teeter moved, while other dogs moved throughout the movement of the falling teeter. Biomechanical strategies for handling the dynamic movement are likely variable based on a dog’s physical characteristics (e.g., height, mass, conformation), balance and coordination, comfort level with movement, and training techniques. Dogs must maintain the coordination needed to compensate for the movement of the obstacle. It is possible that dogs who move through the movement of the teeter have increased balance and coordination, allowing them to compensate for the additional instability and movement. It is also possible that these dogs have more comfort with movement, which could be related to overall temperament or their balance and coordination abilities. Assessing weight shift during movement may be beneficial in further evaluating how dogs handle the movement of the teeter, but weight shift could not be assessed in this study. It is unknown how training contributes to this coordination and comfort with movement. Future work should evaluate correlations with obstacle training strategies as well as relation to a dog’s overall balance and coordination ability.

The time of the descent phase was strongly associated with dog mass, with heavier dogs having a faster teeter descent (Figure 8B). This was not surprising given the physics of the teeter, but it is important to note that teeter drop speed is not equivalent to, or a result of, dog speed. Drop speed is dependent on dog mass and the physics of the lever arm. A faster dog may have a faster overall time to completion due to their ability to get past the fulcrum point faster, but dog speed does not affect the actual rate of descent of the teeter. Also, even with increasing mass, there will be a mechanical limit to the time of descent, due to friction and the design of the teeter, which has speed-limiting, cushioning cylinders on the base to reduce plank whip and rebound (33). In this study, four dogs had teeter descent times less than 0.55 s, but none were less than 0.50 s, suggesting that dogs are approaching the minimum teeter descent time for this teeter. The theoretical limits for the brand of teeter used in this study, which would be tested with a very large weight placed on the very end, is unknown. Observed dog time to descent was generally slower than teeter time to descent, reflecting the lag between the dog’s nose passing the fulcrum of the teeter but before mass is applied to the plank (Table 1). The timing of the stride crossing the fulcrum could affect this relationship, as a paw may precede the nose passing the midpoint. Further work to analyze weight distributions at the time of teeter tipping and throughout the descent phase would provide additional data on how dogs utilize their weight to optimize performance.

### 4.5 Exit

A consistent performance where the dog remains on the teeter until it has contacted the ground, such as using a trained stopped contact behavior, is critical. Failure to successfully perform this obstacle results in a course fault and may potentially result in injury to the dog. The majority of dogs (*n* = 13, 65%) exhibited a stopped contact behavior with 11 of these 13 being a 2o2o behavior with front feet on the ground and rear feet on the teeter. In this study, 1 dog exhibited a 4o standing behavior with all four paws on the teeter plank after touching the ground. A “3 on 1 off” (3o1o) behavior was exhibited by one dog. Since stopped contact behaviors are trained behaviors, the 3o1o was likely meant to be a 2o2o behavior and was not performed accurately.

This is somewhat consistent with previous research that reported that most dogs had a stopped contact behavior and that the majority of those were 2o2o behaviors (3). However, the actual percentages reported in that study were quite different. The survey by Pechette Markley, et al., reported that almost 90% of dogs had a stopped contact behavior, compared to the 65% in this study (3). Of the dogs in the Pechette Markley et al. survey, 52.7% had 2o2o behavior and 28.7% of the dogs had a 4o standing behavior, compared to 55 and 5%, respectively, in this study (3). The differences between studies may be due to the differences in sample size, with this study having a very small sample size, and population parameters (e.g., breeds, masses, heights, conformations), compared to the survey. It may also be due to the fact that the survey was by handler self-report, rather than observed contact behavior. The differences may also be attributed to the represented breeds in the two studies, as the contact behaviors are likely influenced by the size of the dog. It is also possible that the particular setup for this study influenced the performance behavior and that the contact behaviors noted during this study were not reflective of the dog’s normal contact behavior or how the behavior would be cued in other settings.

There was also variation in duration of stopped contact behaviors. Not surprisingly, dogs exhibiting a stopped contact behavior spent more time in contact with the teeter compared to dogs who did not exhibit a stopped behavior. This variation in holding contact behaviors likely depends on specific training techniques as well as the timing of when the handler releases the dog from the behavior. The duration of contact behaviors is also likely to be influenced by the environment. Since speed of agility course completion determines placement ranking, handlers may be more likely to quick release their dogs or not hold the stopped contact behavior for as long of a duration during competition as they do in training. It is unknown how the study environment may have influenced the duty factor on the teeter or stopped contact hold duration. To better evaluate contact behaviors, studies with larger sample sizes and studies evaluating dogs in a more trial-like agility environment are needed.

Dogs with a stopped contact had more footfalls during the exit phase as compared to dogs without a stopped contact. This may be related to the training techniques and contact behaviors that are used to ensure that dogs have at least part of one paw placed in the down contact zone. It is possible that the training techniques used to train a specific contact behavior, as well as the contact behavior itself, cause the dog to be more careful during this phase, thereby resulting in the dog taking more steps to ensure successful contact behavior completion. Interestingly, dogs who performed a stopped contact were faster by all measures except “Time to Dog Exit.” We expected dogs preparing for a stopped contact behavior would result in a slower performance of the obstacle. However, they were only slower when the time holding the contact behavior was included. Overall, the total obstacle completion time was generally similar between dogs with stopped contacts and those without. This suggests that a quick-release version of stopped contact behavior commonly observed during competitions is comparable to, if not faster than, a non-stopped contact behavior for the teeter obstacle.

Exit strides were also highly variable between dogs. Some of this variability appears to be related to contact behavior, with dogs who had a 2o2o stopped contact placing their paws further from the edge of the teeter plank. As this study focused on single obstacle performance, there was not a specified next obstacle to recreate an exit as seen during competition settings, therefore this behavior was not quantified. Future work looking at teeter performance within courses would provide more insights to variability in entry and exits to the teeter.

### 4.6 Limitations and conclusion

Limitations for this study include a sample size of 20 dogs. While there was a wide variety of breeds and body weights, the sample did not necessarily reflect the most common agility breed distribution, nor did it reflect the within-breed variation seen in many of the popular agility breeds, such as Border Collies. In previous studies, the most common breeds competing in agility were Border Collie, followed by mixed-breed, Shetland Sheepdog, and Australian Shepherd (3). While Border Collies were the most common breed in this study, Labrador Retrievers and Weimaraners were overrepresented compared to the general agility population data.

Because of the small sample size, it was not possible to look at correlations between footfall patterns and performance variables with dog size other than mass (e.g., height, other conformation), or specific training techniques. Since this was an experimental setup, the dog’s performance may also not be a true reflection of the dog’s performance in training or competition. It is possible that performance variables may differ substantially when the teeter is performed in a full course setting at speed. With this particular experimental setup, it was also not possible to evaluate how different approach angles and prior obstacle types and orientations affect teeter performance.

Another limitation is that only a single teeter brand was used in this study. While teeters must meet agility organization specifications, there is still variability in teeter design and specifications between manufacturers and even between different lines within the same manufacturer. For more comprehensive evaluation of performance variables, obstacles from multiple manufacturers should be compared. Another limitation was that no veterinary examination was performed so inclusion relied on the handler reporting that their dog was injury-free. Because handlers may not always be able to identify that their dog has a mild injury, some dogs participating in this study could have had an injury or underlying orthopedic disease that could influence performance variables. Injury history data was also not acquired, and previous injury could also influence obstacle performance.

These data suggest that dogs of different sizes use different biomechanical strategies to perform a dynamic obstacle and that variability in contact behavior results in variation in performance strategies. Results of this study provide insight into teeter performance strategies and variables that can be utilized for evaluation in future biomechanical studies. This study also provides initial data on biomechanical strategies used by dogs on dynamic surfaces, which may offer insight into dynamic stability and postural control in dogs and how that influences injury occurrence during sport. Future studies should recruit a larger number and variety of dogs, making sure to include the most common agility breeds, and a variety of body morphologies within the breeds. Given the notable differences in performance we observed between the smallest and largest dogs, future studies should carefully consider dog size. Future studies should also include more repetitions, camera angles that ensure all data is captured, and should be validated against videos of dogs in a training and competition setting. Future data capturing kinematics and kinetics throughout the performance phases would provide more robust data for clinical and performance correlations. Analysis of the performance of different brands of teeters with specific weights placed at known distances from the fulcrum would provide insight into obstacle variability and allow for theoretical models of optimal dog performance. Collecting details on training history and injury history in a larger population of dogs may allow for correlation between performance variables, training techniques and injury. Results of this study provide foundational context to future biomechanical studies of canines on dynamic surfaces, which may offer insight into sport injury development and prevention.

## Data Availability

The original contributions presented in the study are included in the article/Supplementary material, further inquiries can be directed to the corresponding author.
