# Supplementary material for: Variability in performance of agility dogs navigating a dynamic obstacle
Source: Front Vet Sci. 2024 Dec 4;11:1492391. doi: 10.3389/fvets.2024.1492391 (PMC11652533; doi:10.3389/fvets.2024.1492391)
Supplement: Supplementary file 1 [file Table_1.docx]

**Supplemental Table 1.** Individual data observed including positional data.

| **Mass Category (kg)** | **ID** | **Breed** | **Mass (kg)** | **Dog Height (in)** | **Stopped Contact** | **Total number of footfalls - positional** | **Footfalls on up ramp - contact zone** | **Footfalls on up ramp - not contact zone** | **Footfalls on down ramp - not contact zone** | **Footfalls on down ramp - contact zone** | **Total number of footfalls – duty factor** | **Footfalls ascent** | **Footfalls descent** | **Footfalls exit** |
| --- | --- | --- | --- | --- | --- | --- | --- | --- | --- | --- | --- | --- | --- | --- |
| <10 | IG00_0001 | Italian Greyhound | 4.0 | 13.5 | No | 22 | 4 | 5 | 6 | 7 | 22 | 10 | 10 | 2 |
|  | MIX0_0001 | Mix | 5.0 | 12.5 | No | 24 | 4 | 4 | 11 | 5 | 26 | 13 | 7 | 6 |
| 10-20 | SS00_0002 | Shetland Sheepdog | 10.5 | 15.75 | 2o2o | 20 | 4 | 1 | 4 | 11 | 18 | 5 | 12 | 1 |
|  | MIX0_0003 | Mix | 13.5 | 17.5 | No | 19 | 4 | 2 | 9 | 4 | 19 | 7 | 7 | 5 |
|  | BC00_0003 | Border Collie | 13.6 | 18.0 | 2o2o | 18 | 1 | 3 | 4 | 10 | 19 | 6 | 7 | 6 |
|  | MIX0_0002 | Mix | 15.0 | 17.25 | 4o | 18 | 3 | 1 | 4 | 10 | 18 | 6 | 6 | 6 |
|  | BC00_0005 | Border Collie | 15.3 | 18.0 | 2o2o | 18 | 4 | 1 | 3 | 10 | 20 | 6 | 8 | 6 |
|  | BC00_0006 | Border Collie | 16.4 | 18.0 | 2o2o | 19 | 4 | 3 | 5 | 7 | 21 | 9 | 6 | 6 |
| 20-30 | BC00_0008 | Border Collie | 20.5 | 21.25 | 2o2o | 18 | 4 | 2 | 4 | 8 | 20 | 6 | 7 | 7 |
|  | BC00_0007 | Border Collie | 21.8 | 21.0 | No | 15 | 4 | 1 | 6 | 4 | 14 | 5 | 6 | 3 |
|  | BC00_0004 | Border Collie | 23.1 | 23.0 | No | 17 | 2 | 3 | 5 | 7 | 17 | 6 | 6 | 5 |
|  | LAB0_0003 | Labrador Retriever | 23.6 | 22.0 | No | 12 | 3 | 1 | 4 | 4 | 12 | 5 | 5 | 2 |
|  | LAB0_0004 | Labrador Retriever | 23.6 | 22.0 | 2o2o | 18 | 4 | 1 | 5 | 8 | 23 | 6 | 7 | 10 |
|  | LAB0_0001 | Labrador Retriever | 25.5 | 21.5 | No | 12 | 0 | 4 | 1 | 7 | 12 | 4 | 4 | 4 |
|  | LAB0_0002 | Labrador Retriever | 26.2 | 22.0 | 3o1o | 14 | 3 | 1 | 5 | 5 | 18 | 5 | 4 | 9 |
|  | WEIM_0002 | Weimaraner | 26.4 | 24.5 | 2o2o | 14 | 2 | 2 | 4 | 6 | 17 | 5 | 3 | 9 |
|  | DOB0_0001 | Doberman Pinscher | 27.3 | 24.5 | 2o2o | 18 | 1 | 2 | 6 | 9 | 19 | 4 | 4 | 11 |
|  | DOB0_0002 | Doberman Pinscher | 29.0 | 25.5 | 2o2o | 15 | 2 | 2 | 2 | 9 | 18 | 5 | 3 | 10 |
| >30 | WEIM_0003 | Weimaraner | 30.9 | 24.0 | 2o2o | 15 | 3 | 1 | 5 | 6 | 17 | 5 | 4 | 8 |
|  | WEIM_0001 | Weimaraner | 39.6 | 27.0 | 2o2o | 13 | 0 | 4 | 3 | 6 | 16 | 5 | 2 | 9 |

**Supplemental Table 2.** Individual data observed including timing data.

| **Mass Category (kg)** | **ID** | **Breed** | **Mass (kg)** | **Dog Height (in)** | **Stopped Contact** | **Total obstacle completion time (sec)*** | **Dog time to descent (sec)#** | **Teeter time to descent (sec)†** | **Ascent time (sec)‡** | **Time to dog exit**  **(sec)^** |
| --- | --- | --- | --- | --- | --- | --- | --- | --- | --- | --- |
| <10 | IG00_0001 | Italian Greyhound | 4.0 | 13.5 | No | 1.92108 | 1.4250 | 1.0542 | 0.8667 | 0.1880 |
|  | MIX0_0001 | Mix | 5.0 | 12.5 | No | 2.5500 | 1.9292 | 1.4417 | 1.1083 | 0.4400 |
| 10-20 | SS00_0002 | Shetland Sheepdog | 10.5 | 15.75 | 2o2o | 1.2875 | 0.9292 | 0.9292 | 0.3583 | 0.9920 |
|  | MIX0_0003 | Mix | 13.5 | 17.5 | No | 1.6958 | 1.1750 | 0.8458 | 0.8500 | 0.6720 |
|  | BC00_0003 | Border Collie | 13.6 | 18.0 | 2o2o | 1.0917 | 0.7750 | 0.6333 | 0.4583 | 0.9400 |
|  | MIX0_0002 | Mix | 15.0 | 17.25 | 4o | 1.1333 | 0.7750 | 0.5917 | 0.5417 | 1.2640 |
|  | BC00_0005 | Border Collie | 15.3 | 18.0 | 2o2o | 1.0708 | 0.7417 | 0.6792 | 0.3917 | 1.6160 |
|  | BC00_0006 | Border Collie | 16.4 | 18.0 | 2o2o | 1.2583 | 0.8583 | 0.6292 | 0.6292 | NA |
| 20-30 | BC00_0008 | Border Collie | 20.5 | 21.25 | 2o2o | 1.1458 | 0.7542 | 0.6375 | 0.5083 | 1.0440 |
|  | BC00_0007 | Border Collie | 21.8 | 21.0 | No | 1.4125 | 0.9417 | 0.7458 | 0.6667 | 0.3960 |
|  | BC00_0004 | Border Collie | 23.1 | 23.0 | No | 0.9833 | 0.6625 | 0.5750 | 0.4083 | 0.5840 |
|  | LAB0_0003 | Labrador Retriever | 23.6 | 22.0 | No | 0.9625 | 0.6542 | 0.5958 | 0.3667 | 0.0240 |
|  | LAB0_0004 | Labrador Retriever | 23.6 | 22.0 | 2o2o | 1.1000 | 0.7458 | 0.7833 | 0.3167 | 3.4680 |
|  | LAB0_0001 | Labrador Retriever | 25.5 | 21.5 | No | 0.9917 | 0.6792 | 0.5083 | 0.4833 | 0.2560 |
|  | LAB0_0002 | Labrador Retriever | 26.2 | 22.0 | 3o1o | 1.0792 | 0.6958 | 0.6417 | 0.4375 | 1.3360 |
|  | WEIM_0002 | Weimaraner | 26.4 | 24.5 | 2o2o | 1.4083 | 1.0667 | 0.5125 | 0.8958 | 1.5920 |
|  | DOB0_0001 | Doberman Pinscher | 27.3 | 24.5 | 2o2o | 1.34584 | 0.8333 | 0.5625 | 0.7833 | 2.7680 |
|  | DOB0_0002 | Doberman Pinscher | 29.0 | 25.5 | 2o2o | 1.3625 | 0.7750 | 0.5500 | 0.8125 | 1.9760 |
| >30 | WEIM_0003 | Weimaraner | 30.9 | 24.0 | 2o2o | 1.2125 | 0.7625 | 0.5208 | 0.6917 | 2.3200 |
|  | WEIM_0001 | Weimaraner | 39.6 | 27.0 | 2o2o | 1.1792 | 0.7375 | 0.5292 | 0.6500 | 1.7280 |

* time from when nose crosses teeter threshold until teeter touches the ground

# time from when nose crosses midpoint until teeter touches the ground

† time from when teeter starts to move until teeter touches the ground

**‡** time from when nose crosses teeter threshold until teeter starts to move

^ time from when teeter touches ground to last paw contact
